# Lung-specific interleukin 6 mediated transglutaminase 2 activation and cardiopulmonary fibrogenesis

**DOI:** 10.3389/fimmu.2024.1371706

**Published:** 2024-04-08

**Authors:** Krishna C. Penumatsa, Yamini Sharma, Rod R. Warburton, Adit Singhal, Deniz Toksoz, Chinmayee D. Bhedi, Guanming Qi, Ioana R. Preston, Christina Anderlind, Nicholas S. Hill, Barry L. Fanburg

**Affiliations:** Pulmonary, Critical Care and Sleep Division, Department of Medicine, Tufts Medical Center, Boston, MA, United States

**Keywords:** interleukin 6, transglutaminase 2, PKM2, lung, pulmonary arterial hypertension, right ventricle, fibrosis

## Abstract

Pulmonary hypertension (PH) pathogenesis is driven by inflammatory and metabolic derangements as well as glycolytic reprogramming. Induction of both interleukin 6 (IL6) and transglutaminase 2 (TG2) expression participates in human and experimental cardiovascular diseases. However, little is known about the role of TG2 in these pathologic processes. The current study aimed to investigate the molecular interactions between TG2 and IL6 in mediation of tissue remodeling in PH. A lung-specific IL6 over-expressing transgenic mouse strain showed elevated right ventricular (RV) systolic pressure as well as increased wet and dry tissue weights and tissue fibrosis in both lungs and RVs compared to age-matched wild-type littermates. In addition, IL6 over-expression induced the glycolytic and fibrogenic markers, hypoxia-inducible factor 1α, pyruvate kinase M2 (PKM2), and TG2. Consistent with these findings, IL6 induced the expression of both glycolytic and pro-fibrogenic markers in cultured lung fibroblasts. IL6 also induced TG2 activation and the accumulation of TG2 in the extracellular matrix. Pharmacologic inhibition of the glycolytic enzyme, PKM2 significantly attenuated IL6-induced TG2 activity and fibrogenesis. Thus, we conclude that IL6-induced TG2 activity and cardiopulmonary remodeling associated with tissue fibrosis are under regulatory control of the glycolytic enzyme, PKM2.

## Introduction

Pulmonary arterial hypertension patients and experimental models of pulmonary hypertension (PH) exhibit increased expression of pro-inflammatory cytokines and chemokines in the perivascular regions of their pulmonary arteries (1). Among them, overexpression and synthesis of Interleukin 6 (IL6) in response to tissue injury contributes to hematopoiesis, autoimmunity and inflammation, as well as fibrosis in the face of unresolved inflammation (2). Previous studies have demonstrated that both IL6 (3, 4) and a pro-fibrogenic protein, transglutaminase 2 (TG2) (5) play significant roles in hypoxia-induced experimental PH. However, the interaction of TG2 and IL6 signaling has never been assessed in the lung or heart. Of note, IL6 has been reported to induce TG2 activity in liver cancer (6). TG2 has been shown to promote epithelial to mesenchymal transition of liver cancer cells and cancer progression in an IL6/signal transducer and activator of transcription (STAT) 3 mediated pathway (7). In addition, higher TG2 expression levels have been shown to induce IL6 secretion and cancer cell aggressiveness (8).

Previous data by others have shown that IL6 and other cytokines regulate glycolysis in cancer (9). Importantly, we previously reported that TG2 activity is under the regulation of glycolysis (10), a linear multiple step metabolic process that converts glucose to pyruvate molecules for generation of cellular energy. Augmentation of glycolysis stabilizes hypoxia-inducible factor 1α (HIF1α), which is a known transcriptional factor for TG2 (11). TG2 activity is also increased and plays a role in inflammation-induced systemic hypertension (12). Thus, in the present experiments, we used a lung specific IL6 over-expressing mouse model to gain further insights into the *in vivo* relationships between IL6, a terminal glycolytic enzyme (PKM2), TG2 and cardiopulmonary tissue remodeling in PH. We also then aimed to determine whether IL-6 participates in the regulation of TG2 activity in lung fibroblasts and whether PKM2 participates in IL-6-mediated TG2 activation and fibrogenic remodeling.

## Materials and methods

### Animals

For *in vivo* studies, lung specific IL6 over-expressor (IL6+) transgenic mice breeders were obtained from the University of Pittsburgh (gift from Dr. Stephen Chan). In the IL6+ mice, the Clara cell 10-kD promoter constitutively drives lung-specific IL6 over-expression (3). Age-matched (3 – 4-month-old) IL6+ and littermate IL6- wild-type (WT) control male and female mice were used for the study in accordance with protocols approved by the Tufts University Institutional Animal Care and Use Committee. Mice were housed in the animal facility at Tufts University School of Medicine and provided food and water *ad libitum*. Mice were anesthetized with Ketamine (100 mg/kg) and xylazine (10 mg/kg) and right ventricular systolic pressures were recorded as previously described (13). Mice were then euthanized by exsanguination and lung and heart tissues were isolated. Wet and dry tissue weights were recorded. For dry weights, the tissue was placed in a 60°C incubator for 24 hours and then weighed.

### Genotyping

The IL6+ mice were identified by genotyping mouse DNA by PCR as described previously (3). Briefly, genotyping of mice was performed by PCR using the following primer pair: forward primer 5’-GCAACAAAAAGTGGGTAAATG-3’ and reverse 5’-CTCCAAAAGACCAGTGATGAT-3’. PCR was carried out using the REDExtract-N-Amp PCR kit (MilliporeSigma, Burlington, MA) and the PCR products were resolved on 1.2% agarose gels using the FlashGel System (Cat# 57067; Lonza).

### Reagents

For *in vitro* studies, recombinant human IL6 was purchased from R&D Systems (Minneapolis, MN). TG2 small molecule inhibitor, ERW1041E (Cat# 5095220001) and pyruvate kinase isoform M2 inhibitor, Shikonin (Cat# S7576) were purchased from MilliporeSigma and both were dissolved in dimethyl sulfoxide (DMSO; MilliporeSigma) as described previously (10).

### Hydroxyproline assay

Hydroxyproline in right lung lobes and cardiac right ventricles was measured using high-performance liquid chromatography as previously described (14). For quantitative assessment of fibrogenic remodeling, hydroxyproline measurements are reported as amounts (μg) per wet weight of tissue.

### Histology

Immunohistochemistry was carried out on formalin-fixed and paraffin-embedded lung, left ventricle (LV) and right ventricle (RV) sections following standard methodology. For qualitative assessment of fibrogenic remodeling, lung, and heart (LV and RV) sections were stained with Picrosirius stain (Cat# 26357-02; Electron Microscopy Sciences, Hatfield, PA). All imaging was performed using light microscopy (Nikon Eclipse E800 microscope; Nikon Instruments, Melville, NY) and Spot Imaging software (Sterling Heights, MI).

### RNA isolation and quantitative RT-PCR

Frozen lung and heart tissues were used for total RNA extraction by TRIzol (Invitrogen, Thermo Fisher Scientific, Waltham, MA) according to the manufacturer’s instructions. Briefly, 1μg total RNA was reverse transcribed using a High-Capacity cDNA Reverse Transcription Kit (Thermo Fisher Scientific). Real-time polymerase chain reaction (PCR) analysis was performed using 2x SYBR Green Master Mix (Thermo Fisher Scientific) on an ABI Prism 7900 Sequence Detection System (Thermo Fisher Scientific) as described previously (10). Human and mouse specific primer sets (IDT Technologies, Coralville, IA) were used (10). 18s ribosomal RNA levels were used to normalize the cycle threshold (Ct) values. A ΔΔCt method was used to express the relative quantification of specific genes as previously described (10).

### Protein extraction and Western blot

Frozen tissues were homogenized, and cell culture lysates were lysed in NP-40 lysis buffer (Boston Bio Products, Ashland, MA) in presence of protease and phosphatase inhibitor cocktails (MilliporeSigma). The lysed samples were then separated into two fractions by centrifugation, the soluble supernatant and the insoluble pellet fraction comprising the extracellular matrix (ECM) proteins. Bradford Assay (Bio-Rad Laboratories) was used to quantify the total protein content. An equal amount of protein lysates or the ECM fractions were then reduced in SDS sample buffer (Boston Bio Products) and processed for SDS-PAGE analysis as described (30). For Western blot analysis, antibodies against target protein transglutaminase 2 (TG2; Cat# sc-48387; Santa Cruz Biotechnology, Dallas, TX); fibronectin (Fn; Cat# sc-8422, Santa Cruz Biotechnology); serotonin (Cat# S5545, MilliporeSigma); Stat3-phospho (Cat# 9145; Cell Signaling Technology, Danvers, MA); Stat3 (Cat# 4904; Cell Signaling Technology); pyruvate kinase M2 (PKM2; Cat# 3198; Cell Signaling Technology); type 1 collagen (Col1; Cat# ab34710, Abcam, Boston, MA); α-smooth muscle actin (α-SMA; Cat# sc-32251; Santa Cruz Biotechnology) and β-actin (Cat# 4970, Cell Signaling Technology) were used. Horseradish peroxidase (HRP) tagged secondary antibodies (Santa Cruz Biotechnology) were used for primary antibody target detection. ECL substrate (Thermo Fisher Scientific) was used to visualize the protein bands. Quantitative image analysis with densitometry was performed using Image J software (NIH).

### Cell culture

For *in vitro* studies, primary mouse lung fibroblasts isolated from WT and IL6+ mice by explant culture; de-identified human PA adventitial fibroblasts isolated from healthy donors at the University of Colorado (gift from Dr. Kurt Stenmark) and control human PA adventitial fibroblasts purchased from a commercial supplier (Catalog #3120; Sciencell Research Laboratories) were used. Fibroblasts were grown in fibroblast growth media supplemented with 5% fetal bovine serum (FBS), fibroblast growth factors and antibiotics (Cat. #2331; ScienCell Research Laboratories, Carlsbad, CA) and used at passage 2-4. Cells were maintained at 37°C in a humidified 5% CO_2_ incubator. Cells were serum starved 1 day prior to treatment and maintained in reduced-serum media (0.2% FBS) for the duration of the cell treatment studies.

### Immunocytochemical analysis

Mouse lung fibroblasts were grown on cover slips in fibroblast growth media as described above. Cells were then serum-starved overnight and incubated with sterile phosphate buffered saline (PBS; vehicle) or recombinant human IL6 protein (Cat# 206-IL; R&D Systems). After 24 hours, followed by a brief PBS wash, cells were fixed with 4% formaldehyde (Tousimis, Rockville, MD) and permeabilized with 0.1% Triton X-100 (Thermo Fisher Scientific). Fixed cells were then blocked with 5% bovine serum albumin (BSA) in PBS and incubated overnight at 4^0^C with 1% BSA (diluent; secondary antibody control) or alpha smooth muscle actin antibody (α-SMA; Cat# sc-32251; Santa Cruz Biotechnology) followed by AlexaFluor 555 conjugate secondary antibody (Thermo Fisher Scientific) for 1 hour in blocking buffer (1% BSA). Cover slips were then mounted on to slides using Vectashield Antifade mounting medium with DAPI (Cat# H-1200; Vector Laboratories, Burlingame, CA) and sealed. Nikon Eclipse E800 fluorescence microscope (Nikon Instruments, Melville, NY) equipped with Spot Imaging software (Sterling Heights, MI) was used to visualize and image the stained cells.

### Whole cell and extracellular TG2 activity assay

Human pulmonary artery adventitial fibroblast cells were seeded in fibroblast growth media as described above on a 96-well cell culture plates (MilliporeSigma). Fibroblast growth media was replaced with reduced-serum fibroblast basal media (0.2% FBS). Next day, a TG2 substrate, 50µM biotin cadaverine (N-(5-Aminopentyl) biotinamide, trifluoroacetic acid Salt) (Cat# A1594; Thermo Fisher Scientific) was then added to the wells. After 24 hours, cell plates were washed with PBS and blocked with 1% BSA in PBS for 1 hour. Cells were then incubated with Streptavidin-HRP conjugate (Cat# GERPN1231; Millipore Sigma) in blocking buffer for 1 hour followed by washing with PBS. For extracellular TG2 activity, cultured cell plates were de-cellularized with 0.25M NH_4_OH (MilliporeSigma) in 50mM Tris pH 7.4 for 10 minutes at room temperature as described previously (15). Cells were then incubated with a chromogenic substrate and visualizing reagent, TMB solution (Cat# ab171522; Abcam). After 5 minutes, Stop Solution for TMB (Cat# ab171529; Abcam) was added to each well and the 450nm absorbance was read in a Tecan SPECTRAFluor Plus microplate reader (Artisan Technology Group, Champaign, IL) equipped with Magellan Data Analysis software (Tecan).

### Statistical analysis

Data are expressed as mean ± standard error of the mean (SEM) or as median with inter-quartile range. Statistical analysis was performed by unpaired t-test or analysis of variance (ANOVA) using a *post hoc* Tukey’s multiple comparison test on GraphPad Prism. P value of < 0.05 was considered statistically significant.

## Results

### Lung-specific IL6 over-expression increased right ventricular systolic pressure and hypertrophy in mice

**Figure 1:**
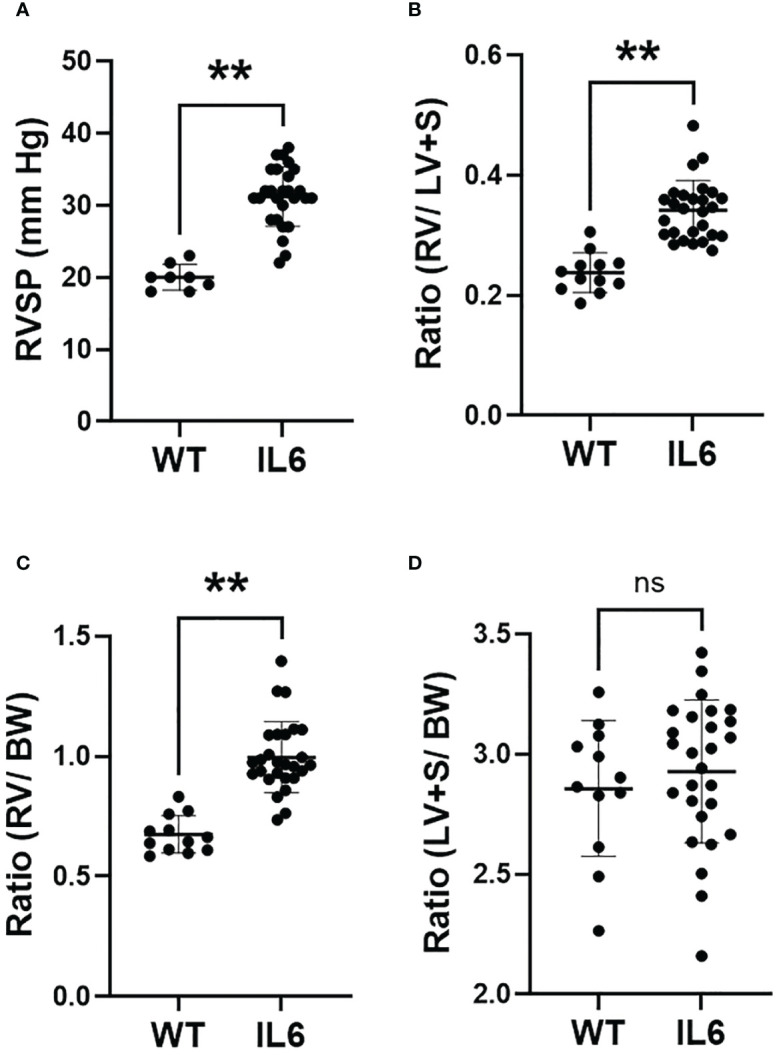
Lung-specific IL6 overexpression induces RVSP and RV hypertrophy. 3 – 4 month old wild-type (WT) and lung-specific IL6 overexpressing (IL6+) transgenic mice were used for the study. **(A)** Scatter plots showing right ventricular systolic pressure (RVSP) measured in anesthetized mice as described in Methods. Cardiac right (RV) and left (LV) ventricular and septal (S) tissue and body weights (BW) were recorded. Scattered plots showing **(B)** Fulton index (RV/LV+S), **(C)** RV/BW ratios and **(D)** LV+S/BW ratios of WT and IL6+ mice. n ≥ 8 animals/group. Statistical analysis was performed by Unpaired t-test. ns, not significant; **p<0.01.

Over-expression of IL6 in mouse lungs significantly elevated right ventricular systolic pressure (RVSP; **Figure 1A**; 31.2 ± 0.79 mmHg), over wild-type mice (20.0 ± 0.62 mmHg), as previously reported (3). Fulton index (RV/LV+S ratio) and ratio of RV/body weights were both significantly greater than in the respective wild-type controls (**Figures 1B, C**). Additionally, the ratio of LV+S/body weights did not differ between IL6+ and WT mice (**Figure 1D**). These results suggest that lung-specific IL6 over-expression enhances both the RVSP and RV tissue remodeling without altering LV+S mass.

### Lung-specific IL6 transgenic mice show increased lung and cardiac right ventricular tissue weights

**Figure 2:**
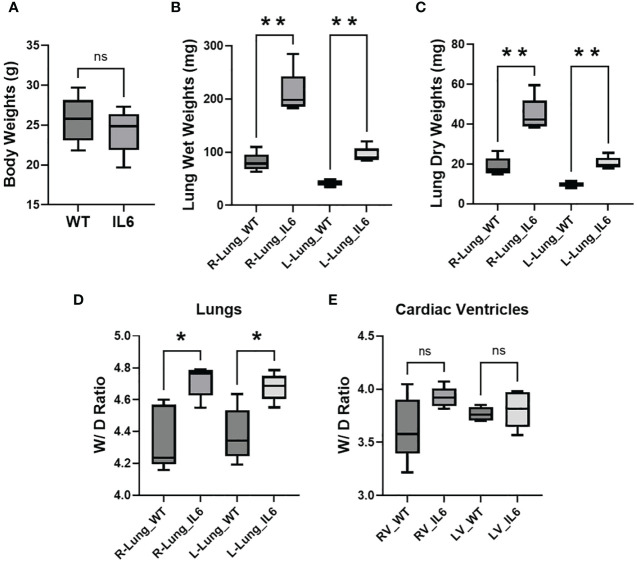
Lung-specific IL6 overexpression increases wet-to-dry weight ratios of lung and cardiac RV tissues. Box plots showing 3-4 month old wild-type (WT) and lung-specific IL6 overexpressing transgenic (IL6+) mouse **(A)** body weights; right (R) and left (L) lung **(B)** wet and **(C)** dry tissue weights; calculated wet-to-dry (W/D) weight ratio of **(D)** right (R) and left (L) lung and **(E)** cardiac right (RV) and left (LV) ventricular tissues. Box plots showing the differences compared to respective WT mouse tissue. n ≥ 5 mice per group. Statistical analysis was performed by Unpaired t-test or ANOVA *post-hoc* Tukey’s test. ns, not significant; *p<0.05; **p<0.01.

Although we found no significant differences in body weights between the IL6+ (25.78 ± 0.97 gms) and WT mice (24.3 ± 0.94gms; **Figure 2A**), we observed that both wet (**Figure 2B**) and dry (**Figure 2C**) lung weights (left and right lung lobes) were significantly increased in IL6+ mice compared to WT littermates by 3 months of age. Furthermore, the ratios of wet-to-dry weights of both left and right lungs were significantly increased (**Figure 2D**). On the other hand, the trends toward increases in cardiac RV and LV wet and dry weight ratios in IL6+ mice were not significantly different (**Figure 2E**) compared to the wild-type littermates.

### IL6+ transgenic mice display increased lung and right ventricular fibrogenic remodeling

**Figure 3:**
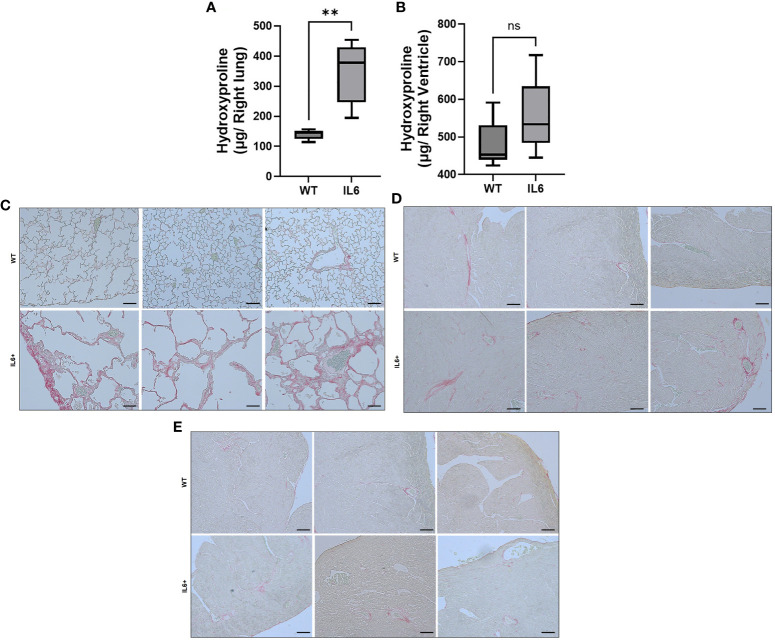
Lung-specific IL6 overexpression increases hydroxyproline content and collagen fibers in lung and cardiac RV tissues. Box plots showing hydroxyproline levels in the **(A)** right lung and **(B)** right ventricle of 3-4 month-old wild-type (WT) and lung-specific IL6 overexpressing transgenic (IL6+) mouse (n≥6 per group). Statistical analysis was performed by Unpaired t-test. **p<0.01; ns, not significant. Picro-sirius red staining was performed to visualize collgen fiber staining and fibrosis in formalin fixed paraffin sections. Representative images of WT and IL6+ mouse **(C)** lung and cardiac **(D)** right and **(E)** left ventruclar tissues were shown. n ≥ 3 mice/group. Representative images were processed identically. Bar = 100 microns.

Lung-specific overexpression of IL6 led to a significant elevation in hydroxyproline levels (**Figure 3A**) in right lung lobes of IL6+ mice (344.45 ± 38.58 µg) compared to those of WT littermate controls (139.45 ± 7.17 µg). Although not significant (p=0.08; **Figure 3B**), we also observed that hydroxyproline levels were moderately elevated in RVs of IL6+ mice (555.53 ± 27.7 µg) compared to WT controls (484.77 ± 22.8 µg).

Interestingly, as seen in **Figure 3C**, the IL6+ lung images show remarkable enlargement of air spaces compared to WT lungs. In addition, we show tissue fibrosis as evidenced by increased collagen accumulation in the left lungs and RVs of lung-specific IL6+ transgenic mice. SiriusRed staining of collagen fibers showed more fibrosis in lungs (**Figure 3C**) and RVs (**Figure 3D**) of IL6+ mice than wild-type littermates. As expected, we did not find a marked increase in collagen staining in the LVs of IL6+ mice (**Figure 3E**).

### Relative mRNA expression of glycolytic and fibrogenic markers are increased in lungs of WT and IL6 transgenic mice

**Figure 4:**
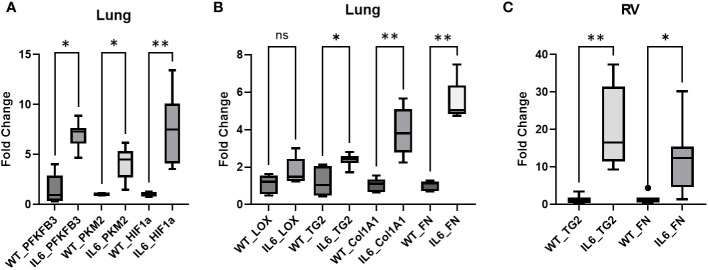
Glycolytic and fibrogenic markers are upregulated in lungs of IL6 transgenic mice compared to age-matched WT control mice. Qunatitative PCR analysis shows the **(A)** mRNA levels of glycolytic markers, 6-phosphofructo-2-kinase/fructose-2,6-biphosphatase 3 (PFKFB3), pyruvate kinease M2 (PKM2) and hypoxia-inducible factor 1α (HIF1α), and **(B)** mRNA levels of fibrogenic markers, lysyl oxidase (LOX), transglutaminase 2 (TG2), collagen 1A1 (Col1A1) and fibronectin (Fn) in lungs of wild-type (WT) control and lung-specific IL6 overexpressing (IL6+) transgenic mice; **(C)** mRNA levels of TG2 and Fn in right ventricles (RV) of WT and IL6+ mice. Box plots showing average fold change in mRNA expression normalized to mouse 18S ribosomal RNA by ΔΔCt method performed in duplicates. n ≥ 3 mice per group. Statistical analysis was performed by ANOVA *post-hoc* Tukey’s test. ns, not significant; *p<0.05 or **p<0.01.

We next investigated if glycolysis participates in IL6 mediated tissue remodeling. Quantitative PCR analysis demonstrated that the rate-limiting glycolytic enzymes, 6-phosphofructo-2-kinase/fructose-2,6-biphosphatase 3 (PFKFB3) and pyruvate kinase isoform M2 (PKM2) and the master transcriptional regulator for glycolytic markers, hypoxia-inducible factor 1-alpha (HIF1α) are markedly up-regulated in the lungs of IL6+ transgenic mice as compared to age-matched WT control mice (**Figure 4A**), suggesting that glycolysis is markedly stimulated by the presence of IL6. Similarly, fibrogenic markers including transglutaminase 2 (TG2), collagen 1A1 (Col1) and fibronectin mRNA levels are elevated in lungs of these IL6+ mice (**Figure 4B**). In addition, although it did not reach statistical significance, there was a trend toward increased mRNA levels of lysyl oxidase (LOX) in lungs of IL6+ mice compared to WT littermates (**Figure 4B**).

### Relative mRNA expression of fibrogenic markers is increased in right ventricles of lung specific IL6 over-expressing mice

To establish the relevance of lung specific IL6 over-expression on RV remodeling, we next evaluated the expression of fibrogenic markers in the RVs of IL6 transgenic mice. As anticipated, fibrogenic markers including TG2 and Fn mRNA expression are significantly increased in RVs of IL6+ mice compared to WT mice (**Figure 4C**).

### Glycolytic markers, TG2 expression and activity and fibrogenic markers are increased in lungs of IL6 over-expressing mice

**Figure 5:**
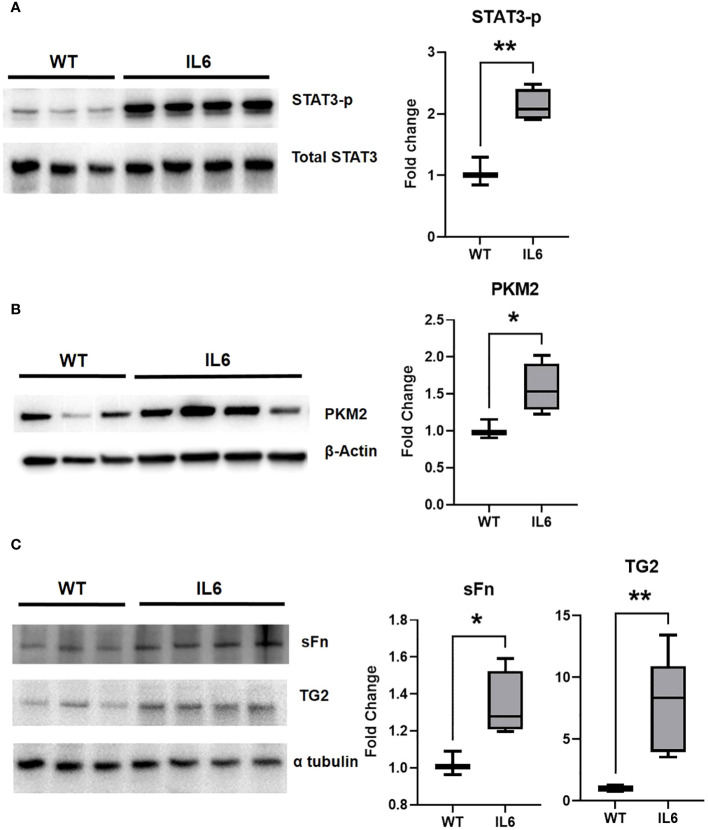
Lung-specific IL6 overexpression induces glycolytic enzymes and TG2 expression and activity in mouse lung tissues. Western blot images showing whole cell lysate expression levels of **(A)** phosphorylated STAT3 (STAT3-p) and **(B)** terminal rate-limitng glycolytic enzyme, pyruvate kinease M2 (PKM2) in lungs of of wild-type (WT) control and lung-specific IL6 overexpressing (IL6+) transgenic mice. Western blot images showing extracellular fraction expression levels of **(C)** serotonylated fibronectin (sFn) and transglutaminase 2 (TG2) in lungs of of WT control and IL6+ mice. Total STAT3, β-actin and α-tubulin were used as loading controls respectively. Box plots showing the normalized fold change differences compared to WT mice. Statistical analysis was performed by Unpaired t-test. n ≥ 3 mice per group. *p<0.05 or **p<0.01.

Next, we investigated the effect of IL6 on STAT3 activation, a canonical transcriptional mediator of IL6 downstream signaling and promoter of pulmonary fibrosis (16). Immunoblot analysis confirmed that the IL6 over-expression increased the ratio of phosphorylated-STAT3/total STAT3 in IL6+ transgenic mouse lungs compared to WT mice (**Figure 5A**). We next confirmed that IL6 over-expression induced the protein expression of glycolytic and fibrogenic markers in the lungs of IL6 transgenic mice as compared to wild-type controls. The lung specific IL6 over-expression induced PKM2 in the mouse lung tissue lysates (**Figure 5B**). In addition, we found that IL6 induced more TG2 expression and activity (sFN levels) in the extracellular matrix fraction of lung tissue lysates of the IL6 transgenic mice than wild-type control mice (**Figure 5C**).

### IL6 induces a profibrogenic phenotype in mouse lung fibroblasts

**Figure 6:**
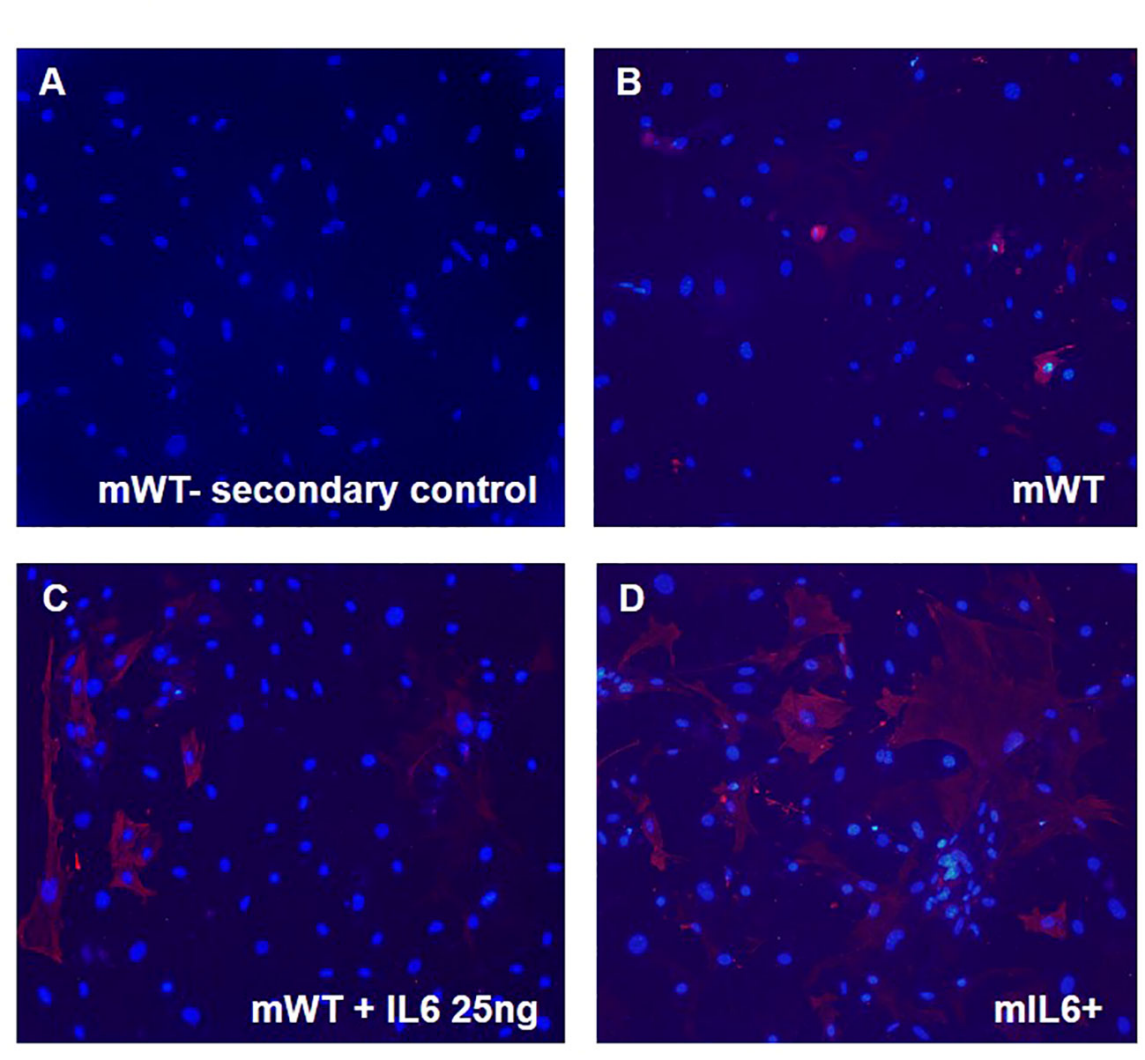
IL6 stimulates myofibroblast transdifferentiation in mouse lung fibroblasts. Lung fibroblast cells isolated from lung-specific IL6 overexpressing transgenic mice (mIL6+) and wild-type control littermates (mWT) were grown on coverslips. mWT lung fibroblasts were treated with vehicle or recombinant IL6 protein [25 ng]. Cells were then imaged after incubation without (secondary antibody control; **A**) and with α-smooth muscle actin [α-SMA; red; **B–D**)] antibody followed by florescent conjugate secondary antibody and nuclear stain DAPI (blue). Shown are representative images for each group (n=3 mouse/group).

To investigate the link between IL6 and fibrogenic remodeling, the induction of a fibroblast cell phenotype was assayed by immunocytochemistry. Lung fibroblasts isolated from both transgenic IL6+ mice and wild-type control mice were grown on coverslips for 72 hours. Wild-type cells were incubated with or without human IL6 recombinant protein. Fibroblasts were then stained with diluent solution (**Figure 6A**) or α-smooth muscle actin (α-SMA) antibody to detect a myofibroblast phenotype (**Figures 6B-D**). Increased expression of α-SMA was evident in the fibroblasts isolated from WT mice treated with recombinant IL6 protein (**Figure 6C**) and IL6+ transgenic mice (**Figure 6D**) compared to the vehicle-treated WT fibroblasts (**Figure 6B**).

### IL6 induced glycolysis and TG2 expression and activity in human pulmonary artery adventitial fibroblasts

**Figure 7:**
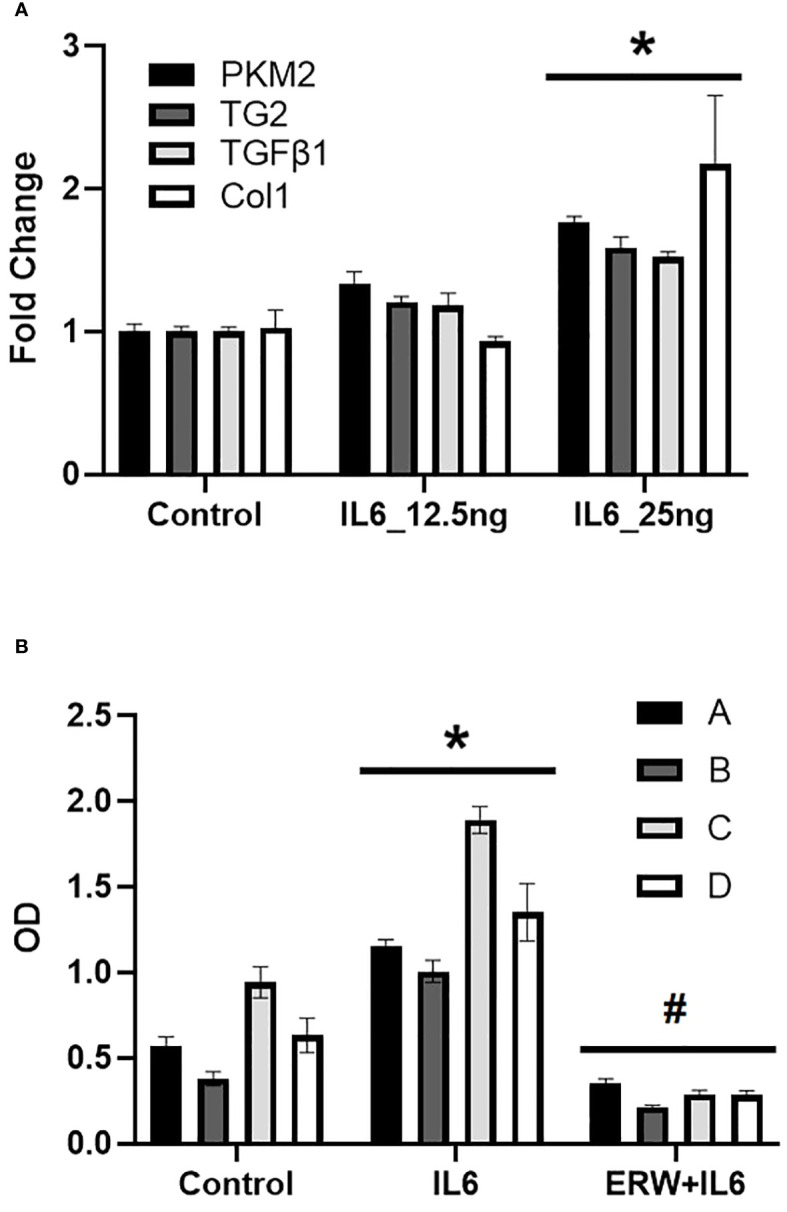
IL6-induced glycolysis, fibrogeneis and TG2 activity in human pulmonary artery adventitial fibroblasts. **(A)** Quantitative PCR analysis demonstrates the effect of concentration-dependent IL6 [12.5 and 25 ng] treatment on pyruvate kinase M2 (PKM2), transglutaminase 2 (TG2), transforming growth factor-β1 (TGFβ1) and collagen 1A1 (Col1) levels in control human pulmonary artery adventitial fibroblasts (hPAAF). Bar graphs demonstrating average fold change in mRNA expression normalized to mouse 18S ribosomal RNA by ΔΔCt method performed in duplicates. Data are presented as mean ± standard error of mean (SEM). *p<0.05; n ≥ 3/group. **(B)** TG2 activity was measured by TG2 substrate, biotin cadaverine [50 µM] incorporation in hPAAF cell lines (n=4) as described in Methods. Bar graphs demonstrating change in TG2 activity in response to recombinant human IL6 [25 ng] treatment ± TG2 inhibitor, ERW1041E (ERW; 50 µM). Data are presented as mean ± SEM. Statistical analysis was performed by two-way ANOVA *post-hoc* Tukey’s test. *Significantly different compared to vehicle control. #Significantly different compared to IL6 treatment. p<0.05.

To investigate a role for IL6 in human pulmonary arterial (PA) remodeling; *in vitro* studies with human pulmonary artery adventitial fibroblasts were carried out to assess effects of IL6 on glycolysis and TG2 activity in human PA adventitial fibroblasts. The results of these studies show that exogenous addition of human recombinant IL6 cytokine induces mRNA expression of glycolytic enzyme and fibrogenic markers in fibroblasts (**Figure 7A**). After stimulation with IL6 for 24 hours, we observed concentration-dependent marked upregulation of PKM2, TG2, TGFβ1 and Col1 mRNA expression (**Figure 7A**). Additionally, we found that IL6 stimulation led to activation of TG2 in four independent primary human PA adventitial fibroblast cell lines (n=4; **Figure 7B**). We further showed that pretreatment with a small molecule TG2 inhibitor significantly blocked the IL6-induced TG2 activity in these primary human fibroblasts (**Figure 7B**).

### IL6-induced PKM2 mediates TG2 activation and fibrogenesis in cultured human pulmonary artery adventitial fibroblasts

**Figure 8:**
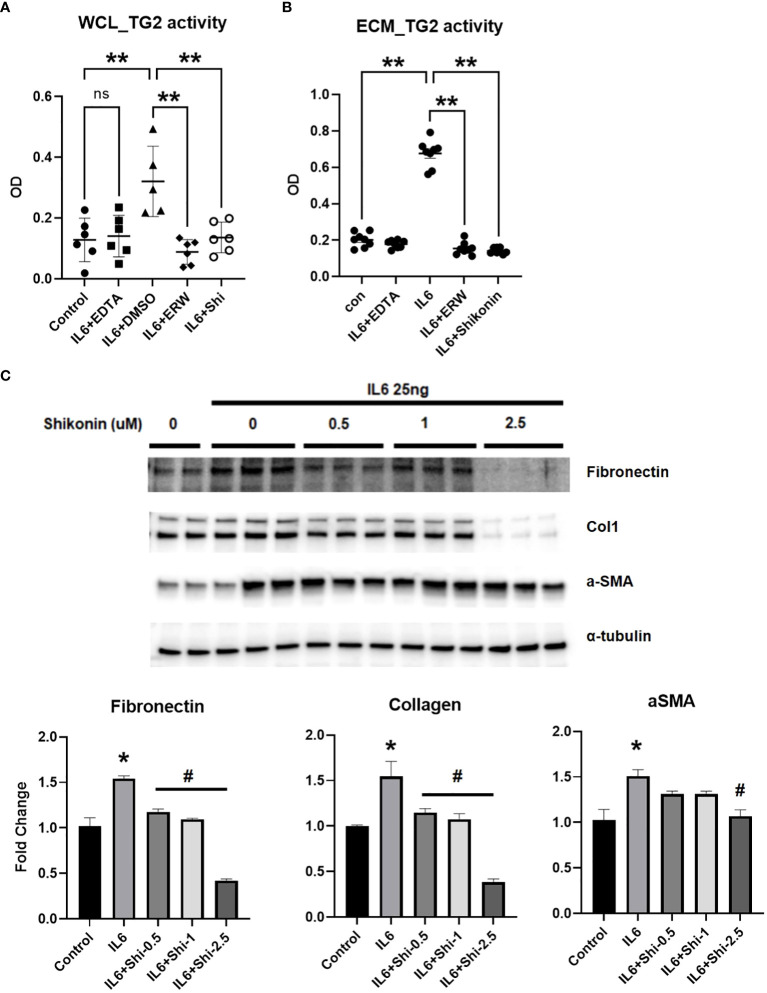
IL6-induced whole cell lysate and extra-cellular matrix TG2 activity and fibrogenesis is mediated by PKM2 in human PA adventitial fibroblasts. Control human pulmonary artery adventitial fibroblasts (hPAAFs; 5000 cells/well) were cultured for 7 days ± IL6 [25 ng] and analyzed for transglutaminase 2 (TG2) activity in both **(A)** whole cell lysate (WCL) and **(B)** extra-cellular matrix (ECM) using TG2 substrate, biotin cadaverine [50 µM] incorporation assay as described in the Methods. Scatter plots demonstrate the IL6-induced TG2 activity in WCL and ECM fraction of hPAAF cells treated with vehicle control (DMSO) or calcium chelating agent, EDTA [1 mM] or TG2 inhbitor, ERW1041E [ERW; 50 µM]) or pyruvate kinase M2 (PKM2) inhibitor, Shikonin [2.5 µM]. Two-way ANOVA *post-hoc* Tukey’s test show significant differences compared to vehicle control/compared to vehicle+IL6. n= 6 replicates/treatment group; ns= not significant; **p<0.01. **(C)** Western blots of protein extracts from hPAAFs pretreated with vehicle control (DMSO) and increasing concentrations [0.5 – 2.5 µM] of PKM2 inhibitor, Shikonin, followed by IL6 [25 ng] treatment for 96 hours showing total Fn (220 kDa) levels, collagen 1A1 (Col1; 130 kDa), α-smooth muscle actin (α-SMA; 42 kDa) levels. Representative of three independent experiments performed in duplicates/triplicates. Bar graphs demonstrate fold change in protein expression normalized to α-tubulin and assessed by densitometry analysis. Two-way ANOVA *post-hoc* Tukey’s test show *significant differences compared to vehicle control group; ^#^significant differences compared to IL6 treatment group. p<0.05.

Previous studies from our lab showed that TG2 is implicated in glycolysis-mediated fibrogenesis in lung and RV fibroblasts (10). To assess the functional role for glycolytic enzyme, PKM2 on IL6-mediated TG2 activation and fibrogenic signaling *in vitro*, we exposed human PA adventitial fibroblasts to IL6 in the presence of vehicle control or Shikonin, a pharmacological inhibitor of PKM2 (17), for 96 hours. IL6 significantly induced TG2 activity in both cell lysates (**Figure 8A**) and the ECM (**Figure 8B**). Furthermore, Shikonin significantly reduced IL6-induced TG2 activity in cell lysates and the ECM (**Figures 8A, B**). Immunoblotting confirmed that the IL6-induced fibrogenesis is mediated by PKM2 in these fibroblasts (**Figure 8C**). We found that pretreatment with Shikonin at varying (0.5 – 2.5 µM) concentrations attenuated the IL6-induced protein expression of fibronectin, Col1 and α-SMA in fibroblasts (**Figure 8C**).

## Discussion

The role of inflammation in the pathogenesis of a variety of cardiovascular diseases is gaining increasing recognition. Not only is there well-established evidence of an inflammatory contribution to systemic vascular disease, but there is also growing evidence for a role in pulmonary vascular remodeling that contributes to RV dysfunction. Our current study suggests that lung-specific overexpression of IL6, a pro-inflammatory mediator, stimulates cardiopulmonary glycolysis, TG2 activation and lung and RV fibrosis, and PH in a transgenic mouse model, in the absence of other triggers of PH.

The pathogenesis of PAH is only partially understood and may vary depending upon whether it falls into the idiopathic or “combined with other diseases” categories. A number of studies have further suggested that PH may be associated with an inflammatory process (1) and/or glycolytic reprogramming (18–20). Of interest, Steiner et al. demonstrated that transgenic lung-specific overexpression of IL6 in normoxic mice induces PH (3). However, the molecular mechanisms involved in IL6-mediated pulmonary and cardiac tissue remodeling in PH pathogenesis remain unclear. In the present study, we confirmed that IL6+ overexpressing mice develop significant increases in RVSP and RV hypertrophy (**Figure 1**), indicative of PH. Previously, Steiner et al. additionally determined that perivascular remodeling occurs in these mice and that this is associated with a significant increase in muscularization of small pulmonary arteries and inflammatory cells in neointimal lesions (3). Our study extends these observations by showing that the lungs from the IL6+ group had a significant increase in both lung wet (**Figure 2B**) and dry (**Figure 2C**) weights compared to lungs from WT mice. Interestingly, we also found that the wet/dry lung weight ratio (**Figure 2D**) was increased indicating that the lung weight increase was significantly associated with water retention (pulmonary edema). In addition, IL6+ over-expressors develop marked increase in hydroxyproline content (**Figures 3A, B**) and collagen accumulation (**Figures 3C, D**), markers of tissue fibrosis in lung and in RVs. These observations support a role for IL6 signaling in regulation of pulmonary hemodynamics and lung and RV tissue fibrogenic remodeling in these animals.

IL6 is known to stimulate aerobic glycolysis (9) and HIF-1α (21) in cancer cells. In addition to its role in hypoxia and anerobic glycolysis, HIF1α mediates aerobic glycolysis by promoting gene transcription (22). In this study, we discovered that the mRNA expression of glycolytic and fibrogenic markers are elevated in the lungs of IL6+ transgenic mice compared to the wild-type littermates. Consistently, the IL6+ mice showed elevations of glycolytic markers in lung tissues, specifically PFKFB3, PKM2 and HIF-1α (**Figure 4A**). Although the increase in mRNA levels of LOX, a copper-dependent matrix protein crosslinking enzyme, did not reach significance, IL6+ transgenic mouse lungs showed a significant upregulation of TG2 mRNA levels (**Figure 4B**). Furthermore, we also found a significant increase in mRNA expression of TG2 in the RVs (**Figure 4C**), suggesting that TG2 may play a significant role in IL6-mediated RV remodeling. Of note, we have previously reported that both molecular knockdown and pharmacological inhibition of TG2 activity significantly attenuates hypoxia- and glycolysis-induced lung and RV fibrogenesis (5). Importantly, we then confirmed that lung-specific IL6 expression induced the phosphorylation of the transcription activator STAT3 (**Figure 5A**), indicating promotion of an inflammation-induced fibrogenic phenotype in these mice. We also identified, for the first-time, that IL6-overexpression induces the protein expression of the terminal rate-limiting glycolytic enzyme, PKM2 (**Figure 5B**) as well as TG2 expression and activity (**Figure 5C**) in mouse lungs. Thus, our prior (5, 10) and current studies suggest that glycolysis and TG2-mediated activities play a key role in induction of downstream fibrogenic tissue remodeling in PH pathophysiology.

Studies from our lab and others (5, 10, 12) have shown that TG2 is implicated in a variety of diseases, including cancer, autoimmune, metabolic, cardiovascular, and fibrotic diseases. TG2 activity is increased and plays a role in inflammation-induced systemic hypertension (12). TG2 is constitutively expressed in the cytosol and is thought to be secreted into the extracellular matrix during fibrogenic progression (23). TG2 catalyzes calcium-dependent post-translational modification of proteins, thereby stabilizing extracellular matrix (ECM) and promoting cellular adhesion and motility functions (24). In addition, TG2 mediates post-translational modification of proteins by calcium-dependent covalent incorporation of serotonin, known as serotonylation (25). Our lab has previously shown that TG2 activity as determined by serotonylation of fibronectin (sFn) is elevated in serum of PAH patients as well as in serum and lungs of experimental rodent models of PH (5, 26). We have also previously reported that glycolysis is most likely the major mediator for TG2 activation in this process (10). Our current study adds to these observations by showing that lung-specific IL6 overexpression induces glycolytic enzyme, PKM2 and activates TG2, and thereby contributes to the development of inflammation-mediated lung and RV tissue fibrosis in experimental PH.

PAH is characterized by an inflammation-induced pro-fibroproliferative process (19). Investigating the molecular mechanisms of IL6, we have found that the addition of exogenous IL6 in cultured fibroblasts induced a profibrogenic phenotype (**Figure 6**). We also found that IL6 increased the expression of PKM2 and TG2 in a concentration dependent manner (**Figure 7**). Thus, as seen in the transgenic IL6+ animal model, a similar profibrogenic phenotype was observed in response to IL6 incubation in cultured mouse and human pulmonary fibroblasts. Also, for the first-time, we report that IL6-mediated TG2 activity (**Figures 8A, B**) and expression of fibrogenic markers (**Figure 8C**) were blocked by inhibition of PKM2, in human PA adventitial fibroblasts. This effect of Shikonin was concentration-dependent, with lower concentrations having minor inhibitory effects on IL6-induction of the downstream fibrogenic markers fibronectin and collagen 1 (**Figure 8C**). However, increasing the Shikonin concentration to 2.5 µM resulted in a robust inhibitory effect on the expression of fibronectin and collagen 1, and a moderate inhibitory effect on α-SMA expression.

Limitations of the current study include the use of pharmacological inhibitors of TG2 (ERW1041E) and PKM2 (Shikonin), which could have off-target effects. However, our previous studies have demonstrated that both effectively inhibit their target’s biological activity in lung fibroblasts (10). Also, we have not tried experiments using inhibition of TG2 or PKM2 in our *in vivo* IL6+ transgenic mouse model, but such experiments in our *in vitro* systems (**Figures 7**, **8**) indicate that both are involved in the regulation of IL-6 induced fibrosis. In addition, cellular systems and experimental models have limitations as representations of human disease. Although inflammation and pro-fibrogenesis are clearly central in the pathogenesis of the human disease, and elevated levels of IL-6 in humans with PAH strongly suggest that it plays a role (27), the precise mechanisms have been elusive. The human disease is characterized by complexity and heterogeneity and as many as 40% of patients do not fit neatly into any one of the five groups used to classify the disease (28). Thus, it is difficult to relate the IL6+ transgenic mouse to any particular group of the human disease, but it appears to have features of Group 1 PAH and Group 3 PH, the latter based on histology images showing lung parenchymal differences (increased fibrotic changes and abnormal enlargement of lung air spaces; **Figure 3C**) from WT mice. Clearly, more studies are needed to further validate the IL6-mediated pathophysiology and underlying mechanisms and pathways.

**Figure 9:**
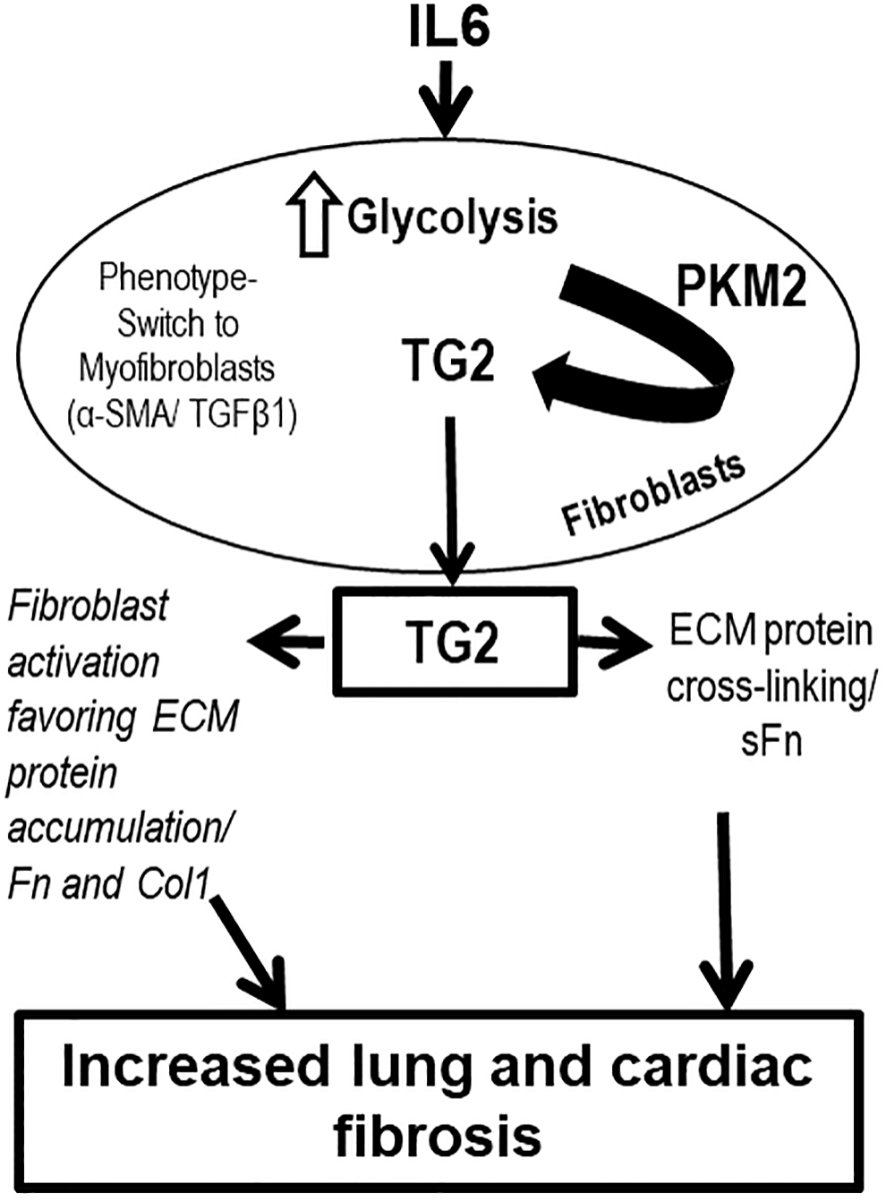
Overall scheme of inflammation-induced glycolysis-mediated TG2 regulation and lung and right ventricular remodeling leading to PH pathogenesis. Inflammation induced by IL6 is associated with increased glycolytic enzyme, PKM2 expression in fibroblast cells. PKM2 regulates TG2 activation and externalization, and mediates ECM protein accumulation and cross-linking. IL6, interleukin 6; PKM2, pyruvate kinase M2; TG2, transglutaminase 2; TGFβ1, transforming growth factor-β1; α-SMA, α-smooth muscle actin; extracellular matrix, ECM; Col1, type 1 collagen; Fn, fibronectin; sFn, serotonylated fibronectin.

In conclusion, our study demonstrates that a lung specific transgenic mouse model that over-expresses IL6 is associated with cardiopulmonary fibrosis and pulmonary hypertension. We also show that the pathway consists of IL6-induced activity of the glycolytic enzyme PKM2, which then activates TG2, leading to fibrosis of the pulmonary vasculature. Human lung interstitial fibroblasts exposed to IL6 increase expression of the fibrotic biomarkers fibronectin and collagen 1, a process that is blunted by inhibitors of PKM2 and TG2, implicating them as key regulators of the process leading to the pro-fibrotic phenotype. This proposed pathway is schematized in **Figure 9**. These findings raise the possibility that TG2 may be a useful therapeutic target in the management of clinical PH and highlight the need for further studies exploring the effects of inflammatory cells and pro-inflammatory cytokines on the glycolytic pathway and TG2-activation in PH pathogenesis.

## Data availability statement

The original contributions presented in the study are included in the article/supplementary material. Further inquiries can be directed to the corresponding author.

## Ethics statement

Ethical approval was not required for the studies on humans in accordance with the local legislation and institutional requirements because only previously established de-identified human cells were used. The animal study was approved by Tufts University Institutional Animal Care and Use Committee. The animal study was conducted in accordance with the local legislation and institutional requirements.

## Author contributions

KP: Project administration, Methodology, Investigation, Funding acquisition, Formal analysis, Data curation, Conceptualization, Writing – review & editing, Writing – original draft, Visualization, Validation, Supervision, Software, Resources. YS: Writing – review & editing, Writing – original draft, Resources, Methodology, Investigation, Formal analysis, Data curation. RW: Writing – review & editing, Writing – original draft, Supervision, Resources, Project administration, Methodology, Investigation. AS: Writing – review & editing, Resources, Methodology, Investigation, Formal analysis. DT: Writing – review & editing, Supervision, Resources, Methodology, Investigation. CB: Writing – review & editing, Resources, Methodology, Investigation, Formal analysis. GQ: Writing – review & editing, Supervision, Resources, Methodology, Investigation. IP: Writing – review & editing, Supervision, Resources, Methodology, Investigation, Conceptualization. CA: Writing – review & editing, Supervision, Resources, Methodology, Investigation, Conceptualization. NH: Writing – review & editing, Writing – original draft, Visualization, Supervision, Software, Resources, Project administration, Methodology, Investigation, Funding acquisition, Data curation, Conceptualization. BF: Writing – review & editing, Writing – original draft, Visualization, Supervision, Software, Resources, Project administration, Methodology, Investigation, Funding acquisition, Data curation, Conceptualization.
